# Ранняя манифестация и прогрессирующее многокомпонентное течение синдрома Маккьюна–Олбрайта–Брайцева у девочки 9 лет: клинический случай и обзор литературы

**DOI:** 10.14341/probl12847

**Published:** 2022-04-30

**Authors:** Я. В. Гирш, М. А. Карева, Н. П. Маказан, Е. Н. Давыгора

**Affiliations:** Сургутский государственный университет; Национальный медицинский исследовательский центр эндокринологии; Национальный медицинский исследовательский центр эндокринологии; Сургутский государственный университет

**Keywords:** синдром Маккьюна–Олбрайта–Брайцева, киста яичника, фиброзная дисплазия, СТГ-гиперсекреция, многоузловой зоб, тиреотоксикоз, гипофосфатемия, гипофосфатемический рахит

## Abstract

Синдром Маккьюна–Олбрайта–Брайцева (синдром МОБ) — очень редкое мультисистемное заболевание, проявляющееся фиброзной дисплазией костей, пятнами цвета кофе-с-молоком, гиперфункцией различных эндокринных желез и рядом патологий других систем организма. Мы приводим описание клинического случая тяжелого прогрессирующего течения синдрома МОБ у девочки 9 лет. С этим диагнозом девочка наблюдается с 2,5 года, когда были выявлены пятна цвета кофе-с-молоком, полиоссальная фиброзная дисплазия, периферическое преждевременное половое развитие на фоне эстроген-секретирующей кисты яичника, многоузловой зоб. В процессе динамического наблюдения отмечалось прекращение активного роста ребенка на фоне деформаций костной системы с множественными повторными переломами конечностей; прогрессирование деформации черепа со стенозом каналов зрительного нерва и ухудшением остроты зрения; развитие СТГ-­гиперсекреции, гипофосфатемического рахита, тахикардии. По каждой из эндокринных дисфункций была назначена соответствующая супрессивная/заместительная терапия. В статье приведены алгоритмы обследования девочки в динамике, критерии выбора покомпонентной тактики ведения и обсуждение особенностей течения всех проявлений синдрома.

## Abstract (en)

McCune–Albright–Braitsev Syndrome (MAB syndrome) is a very rare multisystem disease manifested by fibrous bone dysplasia, coffee-and-milk colored spots, hyperfunction of various endocrine glands and a number of pathologies of other body systems. We present a description of a clinical case of a severe progressive course of MAB Syndrome in a nine-year-old girl. With this diagnosis, the girl is observation of the girl began when she was 2.5 years old, when spots of coffee-and-milk, polyosseous fibrous dysplasia, peripheral premature sexual development against a backdrop of estrogen-­secreting ovarian cysts, multinodular goiter were detected. In the process of dynamic observation, it was noted that the child’s active growth stopped against a backdrop of deformities of the skeletal system with multiple repeated fractures of the extremities; progression of skull deformity with stenosis of the optic nerve canals and deterioration of visual acuity; development of STH hypersecretion, hypophosphatemic rickets, tachycardia. Appropriate suppressive / replacement therapy was prescribed for each of the endocrine dysfunctions. The article presents algorithms for examining a girl in dynamics, criteria for choosing a component-wise management tactics and a discussion of the features of the course of all manifestations of the Syndrome.

## АКТУАЛЬНОСТЬ

Синдром Маккьюна–Олбрайта–Брайцева (синдром МОБ) — очень редкое мультисистемное заболевание, проявляющееся фиброзной дисплазией костей, пятнами цвета кофе-с-молоком, гиперфункцией различных эндокринных желез и рядом патологий других систем организма. Синдром назван в честь врачей Фуллера Олбрайта и Донована Джорджа Маккьюна, которые в 1937 г. впервые описали пациентов с данной симптоматикой [1][2]. За 10 лет до этого, в 1928 г., русский хирург Брайцев Василий Романович впервые представил подробное описание фиброзной дисплазии, в связи с чем в русской транскрипции заболевание носит и его фамилию тоже [3]. Распространенность синдрома МОБ изучена в отдельных странах и составляет от 1:100 000 до 1:1 000 000 [4]. Синдром МОБ обусловлен соматическими мутациями в гене GNAS, кодирующем стимулирующую альфа-субъединицу G-белка (Gas) [5]. G-белки состоят из трех субъединиц (альфа, бета, гамма), их функция — передать сигнал от активированных трансмембранных рецепторов к клеточному ядру. Разновидность альфа-субъединиц G-белка определяет от рецепторов каких гормонов будет осуществляться передача сигнала. Через стимулирующую альфа-субъединицу G-белка в клетках органов-мишеней передается сигнал от паратгормона (ПТГ), тиреотропного гормона (ТТГ), гормона роста-рилизинг-гормона (ГР-РГ), лютеинизирующего гормона (ЛГ), фолликулостимулирующего гормона (ФСГ), адренокортикотропного гормона (АКТГ), меланоцит-стимулирующего гормона (МСГ), глюкагона, вазопрессина, катехоламинов (β-адренорецепторы). Активирующая мутация GNAS приводит к рецептор-независимой активации Gas, вызывая неконтролируемую гиперфункцию различных органов и тканей.

Известными компонентами синдрома МОБ являются пятна цвета кофе-с-молоком, фиброзная дисплазия костей, эстроген-секретирующие кисты яичников у девочек, тестотоксикоз и/или макроорхидизм у мальчиков, тиреотоксикоз и/или многоузловой зоб, СТГ/пролактин-гиперсекреция, врожденный АКТГ-независимый гиперкортицизм вследствие гиперплазии фетальной коры надпочечников, полипы гастроинтестинального тракта, неонатальный холестаз, тахикардия [6–8]. Предугадать тяжесть течения и количество компонентов синдрома невозможно — мутация в GNAS при синдроме МОБ соматическая, т.е. возникает сначала в одной клетке на ранних этапах эмбриогенеза и передается в последующем клонам этой клетки [5][9]. Это приводит к непредсказуемой мозаичности распределения мутантных клеток по органам и системам и высокой вариабельности клинических проявлений.

У синдрома МОБ можно выделить особенности, которые обуславливают трудности при ведении пациентов: возможность различной сочетаемости компонентов с манифестацией в разные периоды жизни, риск тяжелого прогрессирующего течения одного или нескольких компонентов синдрома, сложности определения тактики ведения по каждому из проявлений заболевания, вопросы выбора адекватной медикаментозной терапии и оценки эффективности и безопасности проводимого лечения. Представленное нами наблюдение девочки в течение 7 лет с прогрессирующим течением практически всех известных компонентов может оказаться ценным источником практической информации по этому заболеванию для практикующего врача-детского эндокринолога.

## ОПИСАНИЕ СЛУЧАЯ

Девочка, 9 лет, с 2,5 года находится под наблюдением с диагнозом синдром МОБ.

## Манифестация и основания для постановки диагноза

Впервые девочка была обследована в возрасте 2,5 года в связи с эпизодом кровянистых выделений, сопровождавшихся увеличением молочных желез. Тогда, при первом осмотре, обратили на себя внимание пятна цвета кофе-с-молоком и утиная походка. Пятна отмечались с рождения, утиная походка проявилась с первых шагов в 11 мес. При первичном обследовании по месту жительства были обнаружены повышенный уровень эстрадиола (658 пмоль/л) при допубертатных значениях гонадотропных гормонов (на пробе с бусерелином max выброс ЛГ 0,54 Ед/л, max выброс ФСГ 7,94 Ед/л), опережение костного возраста на 2,5 года (соответствовал 5 годам), эхографические признаки кисты правого яичника, нивелировавшейся к моменту контрольного УЗИ через 2 мес. Тогда же МРТ головного мозга и рентгенография нижних конечностей выявили очаги фиброзной дисплазии (ФД) черепа, бедренных костей, костей голени. Дополнительно при первичном обследовании был обнаружен многоузловой нетоксический зоб: при небольшом общем объеме щитовидной железы (2,7 см3) определялись гипери гипоэхогенные образования, самое большое из которых было 3 см в диаметре.

Классическая триада признаков в виде пятен цвета кофе-с-молоком, периферического преждевременного полового развития на фоне эстроген-секретирующей кисты яичника и полиоссальной ФД позволили установить синдром МОБ; наличие многоузлового зоба также полностью укладывалось в диагноз.

## Алгоритм обследования и определения тактики ведения девочки в динамике

С момента постановки диагноза девочка находится под наблюдением, получая покомпонентное лечение; проводится регулярное обследование с оценкой тяжести течения и скринингом на возможные компоненты заболевания.

## Особенности течения заболевания в динамике по компонентам

Фиброзная дисплазия

С момента выявления ФД носила распространенный прогрессирующий характер. По данным остеогаммасцинтиграфии, в 3,5 года очаги ФД отмечались в костях черепа, в левой плечевой кости, правой подвздошной кости, бедренных костях, костях голени, без видимых деформаций черепа и искривления конечностей. С 4 лет стала заметна прогрессирующая асимметрия костей лицевого черепа, распространялись очаги ФД в конечностях и костях таза. Это привело в дальнейшем к многократным патологическим переломам, укорочению правой ноги, деформациям бедренных костей по типу пастушьего посоха, саблевидным деформациям костей голени, потере способности ходить и значительному искривлению позвоночника, вторичному на фоне патологии нижних конечностей, без очагов ФД в позвонках.

С целью стабилизации прогрессирующей деформации нижних конечностей с 3,5 года девочка перенесла ряд корригирующих остеотомий с металлоостеосинтезом, способствовавших частичному уменьшению разницы в длине ног и выраженности деформаций. На момент описания случая самостоятельная ходьба все еще невозможна, обращает внимание отсутствие роста в течение последних 2 лет. Тактика ведения на данный момент включает плановую дальнейшую оперативную коррекцию имеющихся деформаций. Динамика очагов ФД нижних конечностей и деформация позвоночника представлены на рисунке 1.

**Figure:**
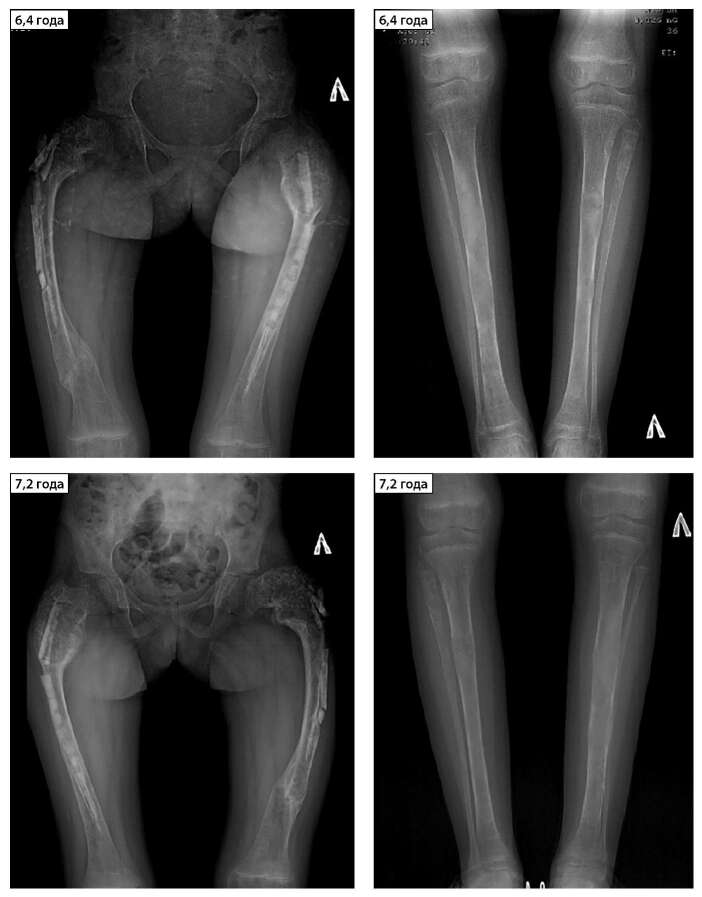
Рисунок 1. Рентгенограммы и компьютерные томограммы нижних конечностей и позвоночника в динамике.Патологические переломы в течение жизни: правой бедренной кости в 4 года, основной фаланги 2 пальца правой стопы в 5,2 года, правой плечевой кости в 5,6 года; правой большеберцовой кости в 5,6 года (через 1 мес после предыдущего); левой бедренной кости в 6 лет. Ортопедические хирургические лечения в течение жизни: внутриочаговая резекция очагов фиброзной дисплазии, металлоостеосинтез левой бедренной кости в 3,6 лет; металлоостеосинтез правой бедренной кости в 4,5 года; удаление металлофиксаторов левой бедренной кости в 5,8 лет (нестабильность металлоконструкции); остеотомия и остеосинтез левой бедренной кости в 8,6 года. Рентгенограммы нижних конечностей в 6,8–9,7 года: прогрессирующая деформация бедренных костей по типу пастушьего посоха, тотальная фиброзная перестройка костной ткани бедренных костей с визуализирующимися металлоконструкциями; очаги фиброзной дисплазии в костях голени с прогрессией в тотальную фиброзную перестройку костной ткани и с формированием саблевидной деформации костей левой голени к 9,2 года. Рентгенограммы позвоночника в 7,8 года: кифосколиоз без очагов фиброзной дисплазии.Figure 1. X-ray and computed tomography of the lower extremities and spine in dynamics.

Помимо деформации ног и вторичных на этом фоне изменений позвоночника и задержки роста, ФД костей привела к асимметрии лица, облитерации придаточных пазух носа и стенозу каналов зрительных нервов (рис. 2). На этом фоне, по результатам недавнего обследования, не отмечено значимого нарушения функции дыхания и вопрос о проведении операций на придаточных пазухах носа (ППН) пока не поднимался. По зрению у девочки отмечается расходящееся содружественное косоглазие, есть гиперметропия слабой степени справа, слева — cмешанный астигматизм и амблиопия средней степени; по данным оптической когерентной томографии (ОКТ) имеется уменьшение толщины слоя нервных волокон слева. Так как не отмечается признаков резкого нарушения остроты зрения и клинических данных за острую компрессию зрительных нервов и/или кровоизлияния в очаги ФД, оперативное лечение по поводу стеноза каналов зрительных нервов также пока не планировалось. Продолжается наблюдение в динамике. Показатели прогрессии и осложнений ФД скелета в целом в динамике представлены в таблице 1. Показатели прогрессии очагов ФД черепа и параметров оценки зрительной функции в динамике подробно представлены в таблице 2.

**Figure:**
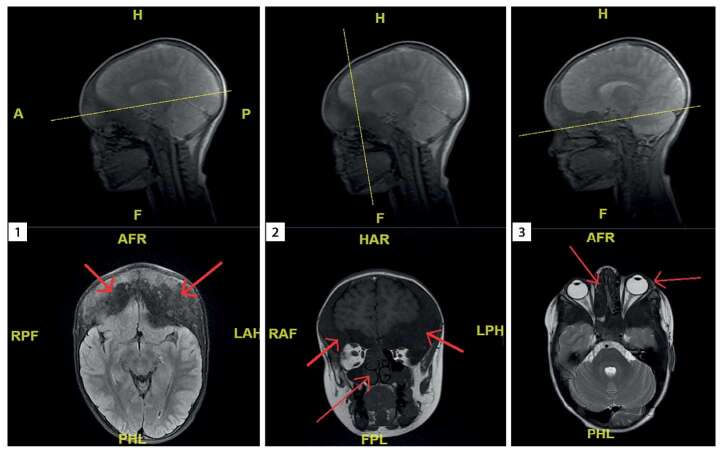
Рисунок 2. МРТ головного мозга в трех срезах.Срез 1 — утолщение лобных костей, облитерация лобных пазух. Срез 2 — утолщение лобных костей, облитерация правой верхнечелюстной пазухи справа, искривление носовых перегородок, сужение носовых ходов. Срез 3 — проптоз глазных яблок, облитерация верхнечелюстной пазухи справа, искривление носовой перегородки.Figure 2. MRI of the brain in three sections.

**Table:** Таблица 1. Фиброзная дисплазия скелета: показатели в динамикеTable 1. Fibrous skeletal dysplasia: indicators over time Сокращения: МСКТ — мультиспиральная компьютерная томография; ЗН — зрительный нерв; ППН — придаточные пазухи носа; ДЗН — диск зрительного нерва; ОКТ — оптическая когерентная томография; КП — компьютерная периметрия; RNFL — retinal nerve fiber layer, средняя толщина слоя нервных волокон по краю диска.

Возраст, годы	ЩФ, ммоль/л	ФД черепа	ФД и деформация конечностей	Деформация позвоночника	Патологические переломы конечностей	Операции на нижних конечностях
2,5			Утиная походка			
3,3	260	Выявлена по сцинтиграфии	Очаги ФД в конечностях выявлены по сцинтиграфии.Утиная походка			
4,5	521	Появилась видимая деформация черепа	Утиная походка		Правой бедренной кости в 4 года.	Внутриочаговая резекция очагов фиброзной дисплазии, металлоостеосинтез левой бедренной кости в 3,6 года.Металлоостеосинтез правой бедренной кости в 4,5 года
5	468	МСКТ: неоднородность структуры костей черепа, с локальными утолщениями отдельных костей; полная и частичная облитерация ППН; сужение каналов ЗН	Первые признаки деформации бедренных костей по типу пастушьих посохов, укорочение правой ноги на 3 см			
5,8	423	Снижение зрения OS	Укорочение правой ноги на 3 см, деформация бедренных костей по типу пастушьих посохов. Прекращение самостоятельной ходьбы		Основной фаланги 2 пальца правой стопы в 5,2 года.Правой плечевой кости в 5,6 года.Правой большеберцовой кости в 5,6 года (через месяц после предыдущего)	Удаление металлофиксаторов левой бедренной кости в 5,8 года (нестабильность металлоконструкции)
6,4	632		Укорочение правой ноги на 3 см усугубление деформации бедренных костей по типу пастушьих посохов.Отсутствие самостоятельной ходьбы		Левой бедренной кости в 6 лет	
7,2	753	МСКТ: отр. динамика по очагам нижней челюсти и ППН.Снижение зрения OD, OS — без динамики	Видимые признаки кифосколиоза		
7,9	977	МСКТ-косвенные признаки компрессии зрительных нервов. Нельзя исключить отек зрительных нервов с обеих сторон. КТ признаки гайморита, сфеноидита справа.ОКТ: OS: истончение нервных волокон.Острота без динамики	Прогрессирующая деформация позвоночника		
8,7	1235	ОКТ: OS: истончение нервных волокон.Острота зрения без динамики	Укорочение правой ноги на 5 см, усугубление деформации бедр. костей по типу пастушьих посохов. Отсутствие самостоятельной ходьбы		Остеотомия и остеосинтез левой бедренной кости в 8,6 года
9,2	1148	ОКТ: OS: снижение толщины нервных волокон.Острота зрения без динамики	Укорочение правой ноги на 3 см.Отсутствие самостоятельной ходьбы		

**Table:** Таблица 2. Фиброзная дисплазия черепа: показатели в динамикеTable 2. Fibrous dysplasia of the skull: indicators in dynamics Сокращения: ЗН — зрительный нерв; ППН — придаточные пазухи носа; ДЗН — диск зрительного нерва; ОКТ — оптическая когерентная томография; RNFL — retinal nerve fiber layer, средняя толщина слоя нервных волокон по краю диска

Возраст, годы	ФД черепа	МСКТ/МРТ	Зрительная функция	СТГ- гиперсекреция, лечение октреотидом
Лицевой череп и ППН	Мозговой череп	Каналы ЗН, мм	Слуховые каналы	Визометрия (Visus)	ЗН ДЗН, ОКТ)	-
3,3	Выявлена по сцинтиграфии							-
4,5	Деформация черепа за счет лобной кости слева					OD 1,0 OS 1,0		-
5	Умер. прогрессия деформации лицевого черепа	(МСКТ) Утолщение лобных костей, больше слева, верхней и нижней верхней челюстей, скуловой кости слева. Умеренная компрессия левой лобной доли утолщенными костями. Левая гайморова пазуха деформирована, уменьшена в объеме, пневматизация сохранена. Правая гайморова пазуха заполнена неоднородным содержимым плотностью до 26 ед.Н. Лобные пазухи не визуализируются	(МСКТ) Глазницы деформированы. На фоне зон повышенной плотности участки пониженной плотности (64 ед.Н). Кортикальный слой кости равномерно истончен на всем протяжении, аналогичные изменения в клиновидной кости, базилярной части затылочной кости, решетчатой, височных костях. Передне-задний размер турецкого седла уменьшен до 5 мм. Гипофиз, шириной 10 мм, высотой 4,4 мм. передне-задний размер до 5 мм. В полости орбит объемных образований не выявлено	D=3,2 S=2,6 (N4-8)	Наружный и внутренний слуховые каналы с обеих сторон не сужены, прослеживаются равномерно.	OD 1,0 OS 1,0	ДЗН: OU бледно-розовый, границы четкие	Выявлена СТГ- гиперсекреция Назначено 10 мг/мес.
5,8	Умер, прогрессия деформации лиц. черепа	(МСКТ) без существенной динамики по сравнению с результатами предыдущего исследования	(МСКТ) без существенной динамики по сравнению с результатами предыдущего исследования			OD 1,0 OS 0,3	ДЗН: OU бледно-розовый, границы четкие	7,5 мг/мес.
6,4	Умер. прогрессия деформации лицевого черепа	(МРТ) Неравномерное утолщение лобной кости	(МРТ) неравномерным утолщение костей основания черепа, лобной и теменных костей. Срединные структуры не смещены. Желудочковая система не расширена и не деформирована. Субарахноидальные пространства не расширены.Турецкое седло уменьшено в сагиттальном размере за счёт костных деформаций. Гипофиз: вертикальный — 6 мм, поперечный — 12 мм, переднезадний — 7 мм	-	-	OD 0,8-0,9 OS 0,3	ДЗН: OU бледно-розовый, границы четкие	7,5 мг/мес
7,2		(МСКТ) по сравнению с результатами предыдущего исследования в 5,8 лет: появление деформации нижней челюсти слева за счет мягкотканого участка размерами до 18,5 мм, плотностью до 20 ед. Н. Утолщение слизистой оболочки левой гайморовой пазухи. Жидкостное скопление в правой верхнечелюстной пазухе и правой камере клиновидной пазухи	Очаги ФД костей мозговой части черепа без существенной динамики	D=3,2 S=2,6 (N4-8).	Без отр. динамики	ODO,8 OS 0,3	ДЗН: OU бледно-розовый, границы четкие	10 мг/мес
7,9		(МСКТ) по сравнению с результатами предыдущего исследования в 7,2 лет: правая верхнечелюстная пазуха практически полностью заполнена содержимым, плотностью до 12 ед.Н. КТ признаки гайморита, сфеноидита справа	(МСКТ) по сравнению с результатами предыдущего исследования в 7,2 года: передние и средние клетки решетчатого лабиринта облитерированы с обеих сторон, правая камера основной кости заполнена содержимым плотностью 42 ед.Н. Косвенные признаки компрессии зрительных нервов. Нельзя исключить отек зрительных нервов с обеих сторон.	D на уровне входа 2,5, в средней трети 3,1, на уровне выхода 4 мм. S на уровне входа в канал 2,9, в средней трети 2,7, на уровне выхода 2,8 мм (4–8 мм)	Без отр. динамики	ODO,6 OS 0,3	ДЗН: OU бледно-розовый, границы четкие ОКТ: OD: RFNL N. OS: RFNL истончение в височном квадранте до 40 нм	10 мг/мес.
8,7		(МРТ) асимметричная деформация и утолщение костей черепа, преимущественно лицевого черепа. Выраженная деформация и утолщение лобной кости справа, полная облитерация лобных пазух, субтотальная облитерия верхнечелюстных пазух, больше левой. Деформация глазниц, уменьшения объема глазниц больше слева	Деформация костей основания черепа, правой теменной костей, затылочной кости, тела и крыльев клиновидной кости; субтотальная облитерация пазух основной кости, ячеек решетчатого лабиринта. Глазные яблоки выстоят кпереди, задний контур расположен кзади от межскуловой линии справа на 5,8 мм, слева на 3,6 мм (норма 9,9±1,7 мм)	на уровне верхней глазничной щели D= 3,5, S= 3,5. ЗН не изменен, расширения периневрального пространства не выявлено.	Без отр. динамики	ODO,6 OS 0,3	ДЗН: без динамики. ОКТ: OD RNFL 98 мм, OS RNFL 87 мм	15 мг/мес
9,2						ODO,6 OS 0,3	ДЗН: N ОКТ: OD RNFL 98 мм, OS RNFL 73 мм	

Гонадотропин-независимое преждевременное половое развитие на фоне эстроген-секретирующих кист яичников

Первый эпизод эстроген-секретирующей кисты правого яичника в 2,5 года нивелировался за 2 мес, после чего менструалоподобные выделения отмечались в 2,9 года, и далее в течение года у девочки была ремиссия по эстроген-секретирующим кистам. С 4,3 до 4,9 годат эпизоды менструалоподобных выделений возникали практически ежемесячно. При обследовании на этом фоне в возрасте 4,5 года отмечалось ускорение роста и прогрессия костного возраста (в 4,5 года соответствовал 9 годам). По данным УЗИ малого таза на тот момент определялась киста правого яичника, в анализах отмечался повышенный уровень эстрадиола (427,47 пмоль/л) на фоне допубертатных значений гонадотропинов (ЛГ — 0,216 Ед/л, ФСГ — 0,66 Ед/л). Учитывая частые эпизоды гиперэстрогении с прогрессией костного возраста, была инициирована антиэстрогенная терапия препаратом фулвестрант в дозе 4 мг/кг/мес, которую девочка получает с 4,5 года по настоящее время. Эстроген-секретирующие кисты (рис. 3) яичников периодически рецидивируют, обуславливая эпизоды менструалоподобных выделений. На этом фоне костный возраст спрогрессировал с 9 до 11,5 года за первый год терапии фулвестрантом и далее — без значимой прогрессии с 5,7 года (рис. 4). В таблице 3 можно ознакомиться с динамикой показателей полового развития. Показатели роста в ней намеренно опущены — в данном случае на темпы роста оказывали влияние особенности течения нескольких компонентов заболевания, что подробно разбирается ниже.

**Figure:**
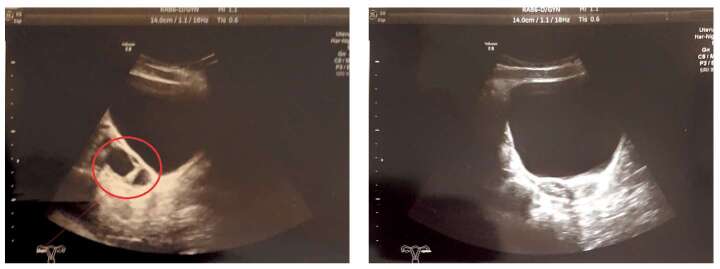
Рисунок 3. УЗИ органов малого таза, выполненное в 9,2 года. В правом яичнике визуализируются две кисты; левый яичник без особенностей.Figure 3. Ultrasound of the pelvic organs, performed at 9.2 years. Two cysts are visualized in the right ovary; the left ovary is unremarkable.

**Figure:**
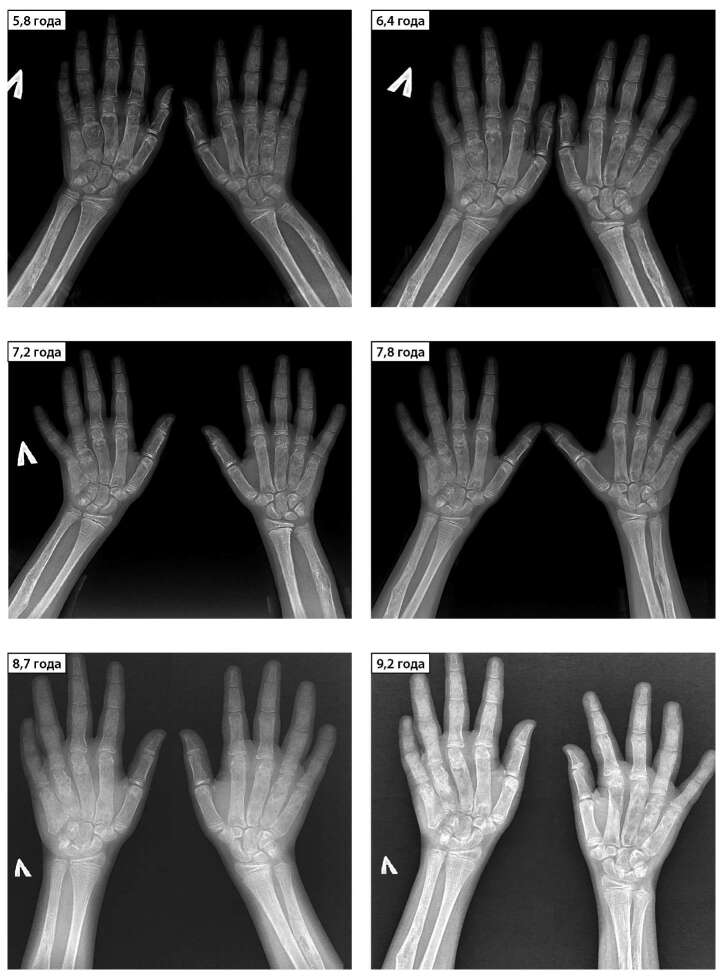
Рисунок 4. Рентгенограммы кистей рук: динамика костного возраста и состояния костей предплечья и кистей рук с 5,8 по 9,2 года. Тотальная фиброзная перестройка пястных костей и фаланг пальцев, очаги ФД и деформация костей предплечья, признаки начала синостоза в концевых фалангах правой руки.

**Table:** Таблица 3. Динамика показателей полового развитияTable 3. Dynamics of indicators of sexual development Сокращения: Э2 — эстрадиол; Ск. роста — скорость роста; пППР — периферическое преждевременное половое развитие; ШМ — шейка матки; ПЯ — правый яичник; ЛЯ — левый яичник.

Возраст в годах	Данные УЗИ ОМТ	Э2 пмоль/л	ЛГ Ед/л	ФС Ед/л	Половое развитие, Tanner	Менструалоподобные выделения на момент обследования	Костный возраст, в годах	Терапия по поводу пППР
2,5	Матка 30х19х30 мм, М-ЭХО 0,34 см. ШМ 26 мм. В ПЯ анэхогенное аваскулярное образование 11 см3. ЛЯ 2 см3 с фолликулами 3-6 мм	657	0,54	7,94		Один эпизод незадолго до исследования	5	-
3,3	Матка 40 ммх12 ммх20 мм, М-ЭХО 0,1 мм. ПЯ 1,5 см3, ЛЯ 1,8 см3	58,9			PI BII	В 2,5 года и в 2,9 года	6,5	-
4,5	Матка 35 ммх20 ммх23 мм, М-ЭХО 0,2 мм. ШМ 24 мм. ПЯ: жидкость-содержащее образование 5,2 мл, ЛЯ 1,7 см3	427,5	0,216	0,66	B2 P1	В 2,5 г, 2,9 лет, с 4,3 года до 4,9 года — практ. ежемесячно	9 лет	Фулвестрант 4 мг/кг/мес
5	Матка 39 ммх24 ммх15 мм, М-ЭХО 0,3 см, ПЯ представлен жидкость-сод. двухкамерным образованием 7,8 мл, ЛЯ 0,8 см3 с фолликулами до 0,5 см	282	0,216	0,66	B2 P1	В 2,5 г, 2,9 лет, с 4,3 года до 4,9 года — практ. ежемесячно.	11,5	Фулвестрант 4 мг/кг/мес
5,8	матка 25 ммх19 ммх12 мм, М-ЭХО 0,2 см, ПЯ 3 см3 с фолликулом 0,8 см. ЛЯ: 1,1 см3 с мелкими фолликулами	69	0,216	0,66	B2 P1	В 2,5 г , 2,9 лет, с 4,3 года до 4,9 года — практ. ежемесячно, с 4,9 до 5,8 года не отмечалось.	11,5	Фулвестрант 4 мг/кг/мес
6,4	Матка 43х24х15 см, М-ЭХО 0,2 см, ПЯ 3,0 см3, фолликулы д до 0,6 см. ЛЯ 2,7 см3 с фолликулами до 0,7 см	88,6	0,216	0,66	B2 P1	В 2,5 г, 2,9 лет, с 4,3 года до 4,9 года — практ. ежемесячно, в 6,1 года однократно	11,5	Фулвестрант 4 мг/кг/мес
7,2	Матка 42х25х18 мм, М-ЭХО 0,2 см. ПЯ представлен жидкость-содержащим образованием размерами: 3,9х2,5х1,8 см. ЛЯ 0,6 см3 с фолликулами д до 0,6 см	204,4	0,216	0,66	B 2, P 2	В 2,5, 2,9 года, с 4,3 до 4,9 года практически ежемесячно, в 6,1, в 6,6, в 7,1 года	11,5	Фулвестрант 4 мг/кг/мес
7,9	матка 41х24х18 мм, М-ЭХО 0,2 см. ПЯ представлен жидкость-содержащим образованием размерами: 2,1х2,0х1,9 см. ЛЯ 2,8 см3 с фолликулами диаметром до 0,6 см	101,3	0,216	3,4	B 2, P 2	В 2,5, 2,9 года, с 4,3 до 4,9 года практически ежемесячно, в 6,1, в 6,6, в 7,1, в 7,3 года	11,5	Фулвестрант 4 мг/кг/мес
8,7	матка 30х24х17 мм, М-ЭХО 0,3 см. ПЯ 9,6 см3, определяется жидкость-содержащее образование 4,2 мл. ЛЯ 6,2 см3, определяется жидкость- содержащее образование 2,0х0,7 см	427,2	0,216	0,66	B 2, P 2	В 2,5, 2,9 года, с 4,3 до 4,9 года практически ежемесячно, в 6,1, в 6,6, в 7,1, в 7,3, в 8,1 года	11,5	Фулвестрант 4 мг/кг/мес
9,2	матка 4х28х16 мм, М-ЭХО не определяется, ПЯ 6,4 см3, в нем два жидкость-содержащих образования диаметром 2,5 см и диаметром 1,5 см. ЛЯ объем 4,1 см3, с фолликулами диаметром до 0,5см	251,9	0.216	1,4	B 2, P 3	В 2,5 г, 2,9 лет, с 4,3 до 4,9 года практически ежемесячно, в 6,1, в 6,6, в 7,1, в 7,3, в 8,1, в 8,7 года	11,5	Фулвестрант 4 мг/кг/мес

Гиперсекреция СТГ

С начала наблюдения базальный уровень СТГ определялся в пределах 1–2 нг/мл при ИФР-1, варьировавшем от 132 до 278 нг/мл со значениями SDS ИФР-1 от +1 до +3 соответственно. Повышенная секреция СТГ была подтверждена в 5 лет по результатам пробы с глюкозой на подавление СТГ, и с 5,3 года девочка получает аналог соматостатина длительного действия (октреотид). Начальная доза составляла 10 мг/мес. При контрольном обследовании через 6 мес, в 5,8 года, обращало внимание выраженное снижение темпов роста, уровень ИФР-1 составлял 82,7 нг/мл, базальный СТГ был 0,74 нг/мл. Доза препарата была снижена до 7,5 мг/мес. На этом фоне в течение следующего года скорость роста улучшилась. При обследовании в 7,2 года вновь отмечалось уменьшение темпов роста, усугубилась деформация черепа, отсутствовало подавление СТГ на пробе с глюкозой. В связи признаками декомпенсации по СТГ-гиперсекреции доза октреотида была вновь повышена, сначала — до 10 мг/мес, а в последующем, к 8,6 года, по тем же показаниям, — до 15 мг/мес. По данным МРТ головного мозга в динамике признаков аденомы гипофиза не отмечается.

За весь период наблюдения темпы роста у девочки варьировали: в некоторые периоды рост был в пределах нормы, периодически темпы роста снижались до 0,5 см/год, и с 7,2 года значимой прибавки в росте не отмечается. В таблице 4 приводятся сводные данные в динамике всех факторов, способных оказывать влияние на скорость роста.

**Table:** Сокращения: Э2 — эстрадиол; Ск. роста — скорость роста; МСКТ — мультиспиральная компьютерная томография; ЗН — зрительный нерв; ППН — придаточные пазухи носа; ОКТ — оптическая когерентная томография, стала доступна к выполнению с 7,9 лет

Возраст, годы	Показатели роста	Гипер- фосфатурия	Гипер-эстрогенемия	СТГ-гиперсекреция	Фиброзная дисплазия (ФД)
Рост2, см	SDS	Ск. роста см/год	SDS ск. роста	P3, ммоль/л	TRP3, %	Э2, пмоль/л	Костный возраст	Фулвестрант	ИФР-1, нг/мл	СТГ, нг/мл	Min СТГ на ОГТТ	Октреотид	ФД черепа	Патол. переломы ниж. кон.	Операции на ниж. кон.	Деформация позвоночника
2,5							657	5	-	-	2,2	-	-				
3,3	100,7	1,6			1,6	82	58,9	6,5	-	132,7	-	-	-	Выявлена по сцинтиграфии	-	-	-
4,5	112	1,72	10,5	2,8	1,4	89	427,5	9	4 мг/мес	277,5	1,6	-	-	Появилась видимая деформация черепа	правой бедр. кости в 4 года	на левой бедр. кости в 3,6 года. На правой бедр. кости в 4,5 года	-
5	114,5	1,2	2,5	-3,6	1,4	89	282	11,5	4 мг/мес	206,7	3,5	1,2	10 мг/мес.	МСКТ: неоднородность структуры костей черепа, утолщения отдельных костей; облитерация ППН; сужение каналов ЗН			
5,8	115	0,67	0,5	-5,44	1,2	76	69	11,5	4 мг/мес	82,7	0,7	-	7,5 мг/мес.	Снижение зрения OS	пальца правой стопы в 5,2 лет. правой плеч. кости в 5,6 лет. правой больше-берцовой кости в 5,6 года	на левой бедренной кости в 5,8 года	-
6,4	118	0,18	5,3	-0,84	1,3	67	88,6	11,5	4 мг/мес	139,8	3,8		7,5 мг/мес.		левой бедренной кости в 6 лет		-
7,2	121,5	0,24	3,7	-2,4	1,2	77	204,4	11,5	4 мг/мес	234	0,6-6,74	1,1	10 мг/мес.	МСКТ: отр. динамика по очагам нижней челюсти и ППН. Снижение зрения OD, OS — без динамики			Появление признаков кифосколиоза
7,9	122,9	-0,13	2,5	-3,79	1,1	71	101,3	11,5	4 мг/мес	177,7	3,1	0,5	10 мг/мес.	МСКТ-косвенные признаки компрессии зрительных нервов, отек ЗН(?) с обеих сторон. ОКТ: OS: истончение нервных волокон. острота зрения без динамики			Прогрессирующая деформация позвоночника
8,7	122,9	-1,15	-	-	0,99	71	427,2	11,5	4 мг/мес	280,8	3,4	-	15 мг/мес	ОКТ: OS: истончение нервных волокон. Острота зрения без динамики		на левой бедренной кости в 8,6 лет
9,2	122,9	-0,7	2	-5	0,94	80	251,9	11,5	4 мг/мес	165,4	1,02	-		ОКТ: OS: снижение толщины нервных волокон. Острота зрения без динамики		

Патология щитовидной железы

При первом обследовании в 2,5 года у девочки был выявлен правосторонний многоузловой эутиреоидный зоб. В динамике с 4,5 года отмечалась тенденция к снижению свободного тироксина (Т4св) при нормальных показателях ТТГ и свободного трийодтиронина (Т3св). В 7,8 года был установлен вторичный гипотиреоз и инициирован прием левотироксина натрия. Морфологические изменения в ткани щитовидной железы, по данным УЗИ в динамике, оставались без значимых изменений в течение всего периода наблюдения. В таблице 5 указаны данные морфофункционального состояния щитовидной железы в динамике, на рисунке 5 — УЗ-картина щитовидной железы на момент обследования в 9,2 года.

**Table:** Таблица 5. Морфофункциональное состояние щитовидной железы в динамикеTable 5. Morphofunctional state of the thyroid gland in dynamics

Возраст, годы	Показатели функции щитовидной железы, сыворотка крови	УЗИ щитовидной железы	Медикаментозная терапия по поводу функции ЩЖ	Прием препаратов с потенциальным эффектом на функцию ЩЖ
ТТГ, мкМе/л (0,64–5,76)	Т4св, пмоль/л (11,5–20,4)	Т3св, пмоль/л (3,8–7,2)	Общий объем, см3	Объемные образования
2,5	0,88	12,8		2,7	в правой доле гиперэхогенное образование с четким неровным контуром 29,68х13,1х11,42 мм, гипоэхогенные образования 8,2х2,59 и 6,15х3,41 мм		
3,3	0,53	14,5	6,6	4,4	в правой доле множественные образования диаметром от 0,7см до 1,7см, с четкими контурами, умеренно пониженной эхогенности, с жидкостными зонами		
4,5	0,49	9,1	6,1	4,5	в правой доле в в/3 размерами: 1,0х0,8х0,6 см в в/3–ср/3д 0,6см и диаметром 0,5см; в ср/3–н/3- конгломерат из двух образований размерами: 2,2х1,6х1,0 см, все с четкими контурами, изоэхогенные, с жидкостными зонами, все с выраженной пери- и умеренной интранодулярной васкуляризацией.		
5	0,98	9,5	6,73	6,0	в правой доле в в/3 р. 1,3х0,8х0,7 см, с четкими контурами изоэхогенное; с жидкостными зонами в в/3-ср/3- д. 0,6 и 0,5 см, смешанной структуры, с четкими контурами; в ср/3–н/3–р. 2,5х1,0х1,6 см, конгломерат из двух образований: с четкими контурами, изоэхогенные с жидкостными зонами; при ЦДК все образования с пери- и умеренной интранодулярной васкуляризацией. EU-TIRADS 2		Подтвержден СТГ- гиперсекреция, назначен октреотид 10 мг/мес.
5,8	0,69	11,2	6,1	6,0	в правой доле изоэхогенные образования с жидкостными зонами, с четкими контурами: в в/3 — р. 1,2х0,9х0,9 см, в ср/3–н/3 по задней поверхности — конгломератное, р. 2,5х1,0х0,9 см. также — множественные 0,6–0,8 см. EU-TIRADS 2		Доза октреотида снижена до 7,5 мг/мес. в связи с задержкой роста
6,4	0,73	12	6	7,1	в правой доле в в/3 размерами: 1,6х1,4х1,0 см, с четкими контурами, смешанной структуры, в в/3–ср/3 диаметром 0,6см и диаметром 0,5 см, оба смешанной структуры, в ср/3–н/3 размерами: 2,6х1,4х1,0 см, с четкими контурами, смешанной структуры. EU-TIRADS 2		
7,2	1,1	10,9	6,4	7,7	в правой доле по всей доле определяются множественные анэхогенные образования диаметром от 0,6 см до 1,6 см, часть из них — с тонкими перегородками. EU-TIRADS 2		Доза октреотида увеличен до 10 мг/мес. в связи с декомпенсацией по СТГ- гиперсекреции
7,9	1,1	9,1	5,2	7,0	в правой доле по всей доле определяются множественные анэхогенные образования диаметром от 0,7 см до 1,7 см, часть из них — с тонкими перегородками. EU-TIRADS 2	Инициирована терапия левотироксином натрия 25 мкг/сут	
8,7	0,47	7,9	5,1	8,3	в правой доле по всей доле определяются множественные анэхогенные образования диаметром от 0,8 см до 1,9 см, часть из них – с тонкими перегородками	Левотироксин натрия 50 мкг/сут	Доза октреотида увеличена до 15 мг/мес. в связи с декомпенсацией по СТГ- гиперсекреции
9,2	0,004	10,2	6,2	8,9	в правой доле по всей доле определяются множественные анэхогенные образования диаметром от 0,6 см до 1,8 см, часть из них — с тонкими перегородками	Левотироксин натрия 75 мкг/сут	

**Figure:**
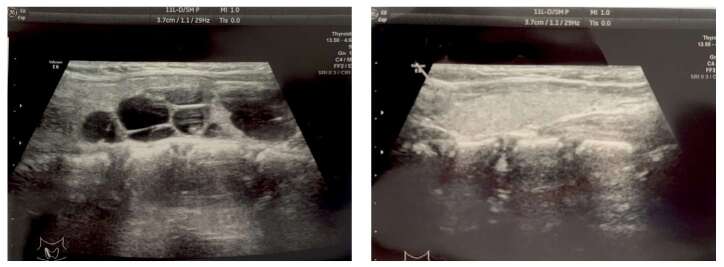
Рисунок 5. УЗИ щитовидной железы, выполненное в 9,2 года. Визуализирована правая доля с множественными анэхогенными образованиями (картина «голландского сыра») и интактная левая доля.

Гиперфосфатурия

Гиперэкскреция фосфора стала отмечаться с 5,8 года. В этом возрасте была инициирована терапия препаратами фосфора в комбинации с альфакальцидолом, которую девочка получала в течение 2 лет. В 7,8 года в связи с эхо-признаками уплотнения чашечно-лоханочной системы и опасениями возможного развития нефрокальциноза терапия фосфором была приостановлена. В динамике уровень фосфора прогрессирующе снижается. В таблице 6 представлены данные по показателям фосфорного обмена в динамике.

**Table:** Таблица 6. Динамика параметров обмена фосфора и лечения гипофосфатемииTable 6. Dynamics of parameters of phosphorus metabolism and treatment of hypophosphatemia

Возраст, годы	Фосфор крови,ммоль/л	TRP, %1	TmP/GFR, ммоль/л2	Препарат фосфора, мг/кг/cут в пересчете на фосфор (+альфакальцидол 0,25 мкг/сут)	УЗИ почек
3,3	1,6(1,10–1,95)3(1,25–2,10)4(0,81–1,94)5	82	1,3(1,15-2,44)6	без терапии	
4,5	1,4(1,00–1,80)3(1,30–1,75)4(0,81–1,94)5	89	1,27(1,15-2,44)6	без терапии	
5	1,4(1,00–1,80)3(1,30–1,75)4(0,81–1,94)5	89	1,18(1,15-2,44)6	без терапии	
5,8	1,2(1,00–1,80)3(1,30–1,75)4(0,81–1,94)5	76	0,88(1,15-2,44)6	35 мг/кг/сут	
6,4	1,3(1,00–1,80)31,30–1,75)4(0,81–1,94)5	67	0,87(1,15-2,44)6	25 мг/кг/сут	
7,2	1,2(1,00–1,80)3(1,20–1,80)4(0,81–1,94)5	77	0,65(1,22-1,6)6	25 мг/кг/сут	двустороннее уплотнение ЧЛС
7,9	1,1(1,00–1,80)3(1,20–1,80)4(0,81–1,94)5	71	0,84(1,22-1,6)6	отмена терапии	двустороннее уплотнение ЧЛС
8,7	0,99(1,00–1,80)3(1,20–1,80)4(0,81–1,94)5	71	0,84(1,22-1,6)6	без терапии	
9,2	0,94(1,00–1,80)3(1,20–1,80)4(0,81–1,94)5	80	0,75(1,22-1,6)6		незначительное 2-стороннее уплотнение внутренней структуры обеих почек

Тахикардия

Периодически у девочки отмечается тенденция к бессимптомной тахикардии без других нарушений со стороны сердечно-сосудистой системы. Терапия адреноблокаторами не назначалась. В динамике частота сердечных сокращений составляла: 112 ударов в минуту в возрасте 2,5 года, 90 ударов в минуту в 5,8 года, 94–96 ударов в минуту в возрастном интервале 7,2–9,2 года.

Текущее состояние

На данный момент у девочки — выраженный кифосколиоз, деформация бедренных костей по типу пастушьих посохов, саблевидная деформация костей левой голени, асимметрия длины нижних конечностей, умеренная деформация костей лицевого черепа, преимущественно за счет левой лобной кости. Тяжелое течение ФД конечностей повлияло на социальную активность и качество жизни — самостоятельная ходьба невозможна, ограничены способности к самоуходу. Облитерация фиброзными массами ППН обуславливает частые рецидивы синуситов с сохранной способностью к носовому дыханию в периоды реконвалесценции. Очаги ФД в мозговой части черепа привели к стенозу каналов зрительных нервов, без КТи МР-признаков компрессии зрительных нервов, но со снижением остроты зрения S>D и уменьшением толщины нервных волокон по краю диска зрительного нерва слева.

Эстроген-секретирующие кисты яичников периодически рецидивируют, для предупреждения преждевременного закрытия зон роста на фоне гиперэстрогении продолжается лечение фулвестрантом в дозе 4 мг/кг/мес. Отмена терапии планируется после достижения паспортного возраста 11–12 лет (с учетом костного возраста). По поводу СТГ-гиперсекреции проводится терапия аналогом соматостатина, доза корректируется по уровню СТГ и ИФР и в настоящее время составляет 15 мг/мес. Лечение СТГ-гиперсекреции пожизненное, отмена терапии чревата усугублением прогрессии фиброзной дисплазии черепа [10]. Многоузловой зоб с умеренным превышением нормы общего объема щитовидной железы и без отрицательной динамики эхо-характеристик узловых образований пока позволяет придерживаться наблюдательной тактики. Гипофосфатемия постепенно нарастает. Тахикардия носит умеренный характер и в настоящее время не требует назначения бета-блокаторов. Продолжается динамическое наблюдение.

## ОБСУЖДЕНИЕ

Манифестация синдрома МОБ в первые годы жизни — нередкая для этого заболевания ситуация. Клинические проявления при рождении и в первые месяцы жизни описаны для пятен цвета кофе-с-молоком, неонатального холестаза, тиреотоксикоза, гиперфункции фетальной коры надпочечников с развитием АКТГ-независимого гиперкортицизма, преждевременного полового развития и фиброзной дисплазии [8][11–13]. В нашем случае на первом году жизни у девочки, скорее всего, уже имелись компоненты синдрома, хотя клинически ярким признаком, выявившим заболевание, стала гиперфункция яичника, спровоцировавшая менструалоподобные выделения и телархе в 2,5 года. Имевшиеся до этого проявления ожидаемо не привлекали к себе внимания: нарушение походки у ребенка первых лет жизни может быть связано с целым рядом более распространенных ортопедических проблем детского возраста, а пятна цвета кофе-с-молоком встречаются среди здоровых детей и расцениваются как значимые, как правило, при выявлении других патогномоничных признаков синдромальной патологии. Заподозрить же у ребенка первых лет жизни многоузловой зоб при эутиреозе и небольшом объеме щитовидной железы было бы еще сложнее.

Таким образом, у девочки основанием для постановки диагноза синдром СОБ стало сочетание периферического преждевременного полового развития и пятен цвета кофе-с-молоком, тогда как имевшиеся в то время другие компоненты — ФД и многоузловой правосторонний зоб — были подтверждены по результатам первого скрининга на возможные проявления заболевания.

После установления диагноза девочке стало проводиться регулярное обследование на все компоненты по алгоритму обследования, разработанному с учетом зарубежных рекомендаций по диагностике и ведению синдрома МОБ, результатов собственного опыта ведения детей с этим заболеванием и с учетом индивидуальных особенностей данного случая [6][14][15]. Скрининг включает исследование ФД, эндокринных гиперфункций и неэндокринных проявлений.

Для диагностики ФД могут использоваться различные визуализирующие методы в зависимости от целей исследования. Остеогаммасцинтиграфия применяется для оценки наличия ФД в случаях, когда диагноз установлен по другим компонентам и неизвестно, вовлечена ли костная ткань в патологический процесс или нет. Также этот метод может быть использован, когда ФД выявлена в одной-нескольких костях с помощью других методов исследования и необходимо оценить наличие процесса в других костях скелета (как это было в описываемом случае). Хотя остеогаммасцинтиграфия успешно определяет все очаги повышенного накопления радиофармпрепарата, давая представление о распространенности процесса ФД, в настоящее время в своей практике мы все больше используем его, скорее, для подтверждения/исключения ФД, чем для оценки распространенности процесса у пациентов с установленной ранее ФД, и применяем этот метод у детей по достижении ими возраста 5–6 лет при отсутствии данных за очаги ФД к этому возрасту по другим методам исследования. Мы пришли к такой тактике по нескольким причинам. Во-первых, остеогаммасцинтиграфия демонстрирует локализацию очагов, но не дает представления об их клинической значимости, а значит, что после выявления очагов в функционально значимых областях (в первую очередь череп, позвоночник, нижние конечности) возникнет необходимость провести дополнительное лучевое обследование, при том что сам метод уже несет лучевую нагрузку. Во-вторых, получение отрицательного результата в первые годы жизни не даст основания исключить ФД как вероятный компонент, так как очаги ФД могут появляться отсроченно, к 5–6 годам [16]. Перспективной альтернативой остеогаммасцинтиграфии может быть МРТ всего тела — метод без лучевой нагрузки и позволяющий оценить очаги ФД с морфологической, а не функциональной точки зрения. Но в любом случае общее исследование всего скелета для решения вопроса о наличии/отсутствии ФД рекомендуется проводить после 5–6 лет [16]. Поэтому, если бы девочка из описываемого нами случая попала на обследование впервые сейчас, в возрасте 2,5 года, то тактика по диагностике ФД немного бы отличалась. В первую очередь было бы проведено исследование угрожаемых по переломам костей скелета, где наличие или отсутствие очагов ФД может повлиять на тактику ведения: нижние конечности, позвоночник и череп. Исследование костей таза, нижних конечностей и позвоночника провели бы с помощью рентгенографии в прямой проекции, тогда как МРТ головного мозга (под наркозом с учетом возраста) или мультиспиральная компьютерная томография (МСКТ) черепа были бы проведены только в случае видимых деформаций черепа, и/или данных за патологию зрения и/или слуха, и/или подозрение на облитерацию ППН по жалобам и результатам осмотра соответствующих специалистов.

Для оценки очагов ФД черепа в последнее время мы стали чаще применять МРТ головного мозга: по этому методу можно определить присутствие очагов ФД, вовлеченность в патологический процесс каналов зрительных нервов, слуховых каналов и костей внутреннего и среднего уха, оценить компрессию зрительных нервов, наличие аденомы гипофиза при СТГ-гиперсекреции. МР-ангиография также может иметь большое значение в случаях ФД черепа для своевременного выявления аномалий сосудов на фоне ФД, угрожающих по развитию кровоизлияний [17]. МСКТ, с другой стороны, уже детально описывает локализацию и распространенность очагов ФД и необходима в случае решения вопроса об оперативном вмешательстве на костях лицевого или мозгового черепа. Вопрос об оперативном лечении рассматривается челюстно-лицевыми и/или нейрохирургами при значимых нарушениях функций носового дыхания, слуха, острых и/или быстро прогрессирующих нарушениях зрения на фоне компрессии каналов зрительных нервов, быстрой прогрессии очагов с формированием социально неприемлемых деформаций, острых состояний при кровоизлияниях на фоне ФД [18][19].

Гиперфункция гонад — частый признак синдрома МОБ [6][14]. У девочек она проявляется периодическим возникновением эстроген-секретирующих кист яичников, провоцирующих эпизоды выделений из половых путей и увеличения молочных желез с обратным развитием [6][14]. Кисты, как правило, нивелируются самостоятельно в течение нескольких недель. Пока есть киста яичника — повышен уровень эстрогенов и есть проявления полового развития, как только киста нивелировалась — признаки полового развития также сходят на нет. Такое волнообразное течение преждевременного полового развития — характерная особенность синдрома МОБ, которая отмечалась и в описываемом нами случае. При выявлении первого эпизода эстроген-секретирующей кисты яичника в 2,5 года была выбрана наблюдательная тактика, так как оставалось неизвестным, будут ли отмечаться у девочки частые рецидивы кист в будущем, что потребовало бы назначения антиэстрогенной терапии, или же эпизоды гиперэстрогении будут возникать достаточно редко, чтобы не иметь клинического значения (то есть не оказывать значимого влияния на темпы прогрессии костного возраста и конечный рост). Уже к 4,5 года стало очевидно, что гиперфункция яичников носит активный характер, обуславливая частые эпизоды повышения эстрадиола крови и прогрессию костного возраста на этом фоне. Такое активное течение периферического преждевременного полового развития (пППР) — с прогрессирующим опережением костного возраста на фоне частых эпизодов гиперэстрогенемии — явилось показанием для назначения антиэстрогенной терапии. [15]. К сожалению, отсутствует таргетная терапия, которая предотвратила бы появление эстроген-секретирующих кист, но есть возможность и даже выбор патогенетического лечения с целью торможения темпов прогрессии костного возраста и улучшения конечного роста.

В арсенале антиэстрогенной терапии, применяемой при синдроме МОБ у девочек, в настоящее время можно выделить две группы препаратов: блокаторы биосинтеза эстрогена (ингибиторы ароматазы — летрозол) и антагонисты эстрогеновых рецепторов (фулвестрант, тамоксифен). Решая вопрос об антиэстрогенной терапии, мы ориентировались на доступные на тот момент результаты опубликованных зарубежных исследований. На момент назначения лечения было доступно больше данных об эффективности и безопасности применения фулвестранта. По летрозолу были данные исследования от 2007 г., проведенные Collins M.T. et al. (National Institutes of Health, NIH, США), согласно которому на фоне лечения улучшался ростовой прогноз, но, с другой стороны, были случаи увеличения в объеме яичников и один случай перекрута кисты на фоне приема препарата (n=9, средний возраст на момент начала терапии 3–8 лет, продолжительность терапии 12–36 мес.) [20]. Тамоксифен является неполным антагонистом эстрогеновых рецепторов — блокирует эффекты эстрогенов на хондроциты и молочные железы, но опосредует их действие на эндометрий [21]. Применение его при синдроме МОБ показало эффективность в виде торможения прогрессии костного возраста, но, с другой стороны, лечение им должно проводиться с осторожностью ввиду риска гиперплазии и перерождения эндометрия [22][23]. По фулвестранту в 2012 г. было опубликовано исследование 30 девочек, получавших терапию в дозе 4 мг/кг/мес в течение года, по результатам которого отмечалось значимое снижение темпов прогрессии костного возраста при отсутствии серьезных побочных эффектов [24]. Исходя из этих данных, для девочки была выбрана терапия фулвестрантом. В настоящее время мы располагаем результатами более длительного исследования эффективности и безопасности летрозола при синдроме МОБ (проведенное также NIH, США), которое позволяет рекомендовать этот препарат при инициации антиэстрогенной терапии по поводу пППР на фоне синдрома МОБ. Исследование было опубликовано в 2016 г. и демонстрировало высокую эффективность летрозола в плане блокирования прогрессии костного возраста без отмеченных побочных эффектов и без изменений по размерам матки и объему яичников (n=28, средний возраст на момент начала терапии 4,6±1,7, средняя длительность терапии 4,1±2,6 года). Ни один из препаратов среди показаний к применению не имеет диагноза пППР, в связи с чем назначение лечения возможно только решением консилиума/врачебной комиссии с участием экспертов федерального уровня.

Оценивая эффективность антиэстрогенной терапии при синдроме МОБ, недостаточно полагаться лишь на темпы прогрессии костного возраста, так как возможным вариантом течения пППР может быть долгий период отсутствия эпизодов гиперэстрогении. В этом случае торможение прогрессии костного возраста и полового развития будет обусловлено не эффективностью лечения, а длительным периодом ремиссии. На протяжении всех 7 лет наблюдения девочке проводилось регулярное УЗИ органов малого таза с оценкой уровня эстрадиола, минимум раз в полгода. За этот период эстроген-секретирующие кисты яичников выявлялись регулярно, тогда как костный возраст перестал прогрессировать через год после начала терапии (см. рис. 4), что мы рассматриваем как доказательство эффективности терапии фулвестрантом.

Динамическое УЗИ малого таза важно не только для девочек, получающих антиэстрогенную терапию, но и для детей без лечения пППР. Во-первых, эпизоды гиперэстрогении могут не сопровождаться яркими проявлениями, когда незначительное напряжение молочных желез и беловатые выделения из половых путей проходят незамеченными. В этих случаях плановое регулярное УЗИ выявит субклинические эстроген-секретирующие кисты, и последующий анализ серии таких исследований даст объективное понимание активности течения пППР. Во-вторых, анализ всех случаев кист яичников в динамике имеет значение при определении показаний к цист/ овариэктомии. Кисты при синдроме МОБ, как правило, быстро нивелируются, но есть случаи их длительной персистенции; регулярное УЗИ позволяет отличить организовавшиеся кисты с низким шансом самостоятельного нивелирования от повторных эпизодов новых кист и решить вопрос о необходимости цистэктомии. В зависимости от распределения мутантных аллелей кисты могут возникать в одном либо в обоих яичниках; при доказанном одностороннем характере гиперфункции есть возможность провести овариэктомию, что может иметь значение для женщин, у которых активная гиперфункция гонады нарушает овуляцию и препятствует зачатию [25]. Показательно, что в описываемом случае более редкое проведение УЗИ могло бы привести к ложному заключению об одностороннем характере процесса: большую часть эпизодов кисты выявлялись в основном в правом яичнике, однако один случай двусторонних кист в 7,9 года опроверг вероятность односторонней гиперфункции гонад у девочки.

Патология щитовидной железы при синдроме МОБ может проявляться многоузловым зобом и/или тиреотоксикозом. У девочки многоузловой зоб определялся уже с 2,5 года жизни, но функциональная активность щитовидной железы никогда не была повышена. Напротив, с 4,5 года у девочки отмечается сниженный Т4св при нормальном уровне Т3св и низком уровне ТТГ, что больше соответствует вторичному гипотиреозу. Можно было бы предполагать, что он развился на фоне приема аналога соматостатина, но все же изменения в тиреоидном профиле стали отмечаться еще до назначения октреотида (табл. 6). Возможно, деформация гипофиза на фоне фиброзной дисплазии могла привести к функциональной недостаточности тиреотрофов, хотя описаний подобного осложнения среди опубликованных случаев синдрома МОБ найдено не было. Эхографические изменения щитовидной железы при синдроме МОБ разнообразны, как правило, доброкачественны, но могут иметь тенденцию к прогрессирующему увеличению в динамике. У девочки множественные кистозные образования привели к формированию характерной для синдрома МОБ эхо-картины «голландского сыра» (см. рис. 5). С момента их выявления не отмечалось значимой отрицательной динамики ни по размерам, ни по эхо-характеристикам образований, в связи с чем продолжается наблюдение за морфофункциональными параметрами щитовидной железы в динамике.

На момент первого исследования в 2,5 года были установлены пППР, многоузловой зоб и ФД. Другие компоненты заболевания были выявлены позднее, однако возраст их диагностики необязательно совпадает с возрастом манифестации этих компонентов. Анализируя данные первого обследования, можно обратить внимание на базальный уровень СТГ, соответствовавший 2,2 нг/мл. Такое значение не исключает, что СТГ-гиперсекреция уже отмечалась в тот момент, но, с другой стороны, и утверждать этого нельзя, не имея результатов СТГ-супрессивного теста и учитывая возможность повышенной секреции гормона роста на фоне отмечавшейся в тот период гиперэстрогенемии [26][27]. Своевременное выявление СТГ-гиперсекреции при синдроме МОБ имеет особенно важное значение в случаях, когда есть очаги ФД в черепе: отсутствие раннего назначения лечения по поводу СТГ-гиперсекреции приводит к прогрессированию ФД черепа с развитием функциональных нарушений, в первую очередь со стороны органов зрения [10][28]. Достоверные данные о наличии СТГ-гиперсекреции у девочки были получены в 5 лет. На тот момент уже имелись видимая деформация черепа и стеноз каналов зрительных нервов (см. таблицу 1). Начальная доза терапии аналогом соматостатина была небольшой — 10 мг/мес — но и ее мы пробовали снижать, когда отметили снижение скорости роста у девочки к 5,8 года. Возможность торможения роста на фоне такой дозы аналога соматостатина еще не встречалась в нашей практике. Кроме того, на темпы роста девочки могли оказать влияние нарушение обмена фосфора и прогрессирующая ФД нижних конечностей. К возрасту 5,8 года уже было 2 перелома костей правой конечности, сформировалась деформация бедренных костей по типу пастушьих посохов с укорочением правой ноги, самостоятельные передвижения стали практически невозможны, уровень фосфора уже показывал тенденцию к снижению (см. табл. 1, 4, 6). Тем не менее влияние октреотида на линейный рост, видимо, все же было, учитывая, что на фоне снижения дозы темпы роста улучшились. Но то же время в динамике отмечалось нарастание уровней ИФР и СТГ, прогрессирование ФД черепа и снижение остроты зрения. Взвесив риски высокой дозы октреотида для роста и ее потенциальную пользу для состояния черепа, был сделан выбор в пользу последнего, и доза была вновь повышена, сначала до 10 мг/мес, а затем и до 15 мг/мес.

Если СТГ-гиперсекреция усугубляет течение ФД черепа, то гипофосфатемия может вносить вклад в состояние очагов любой локализации, как показал ряд исследований, проведенных NIH. Анализ 35 пациентов с синдромом МОБ и изолированной ФД (из них с гиперфосфатурией n=12, с другими эндокринопатиями n=15 и только с ФД n=8) показал, что у детей с ФД и гиперфосфатурией переломы возникают раньше и чаще, чем у детей с ФД без нарушения обмена фосфора [29]. По результатам сравнительного исследования 84 пациентов со сколиозом на фоне синдрома МОБ и 54 пациентов с синдромом МОБ без патологии позвоночника была выявлена значимая разница между степенью тяжести ФД в 2 группах (12 баллов у пациентов без сколиоза и от 34,6 до 64,4 у пациентов со сколиозом) и частотой встречаемости двух компонентов — тиреотоксикоза (с тиреотоксикозом без сколиоза — 20%, с тиреотоксикозом и тяжелым течением сколиоза — 78%) и гипофосфатемии (с гипофосфатемией без сколиоза 11%, с гипофосфатемией и тяжелым течением сколиоза 61%) [30]. Развитие базилярной инвагинации на фоне ФД было отмечено при синдроме МОБ у пациентов, имевших тиреотоксикоз, гипофосфатемию, пППР (пациенты с ФД черепа — n=158, из них базилярная инвагинация — n=12). Кроме того, гипофосфатемия может обусловить мышечную слабость, болевой синдром, снижение темпов роста.

С одной стороны, приведенные данные демонстрируют клиническую значимость гипофосфатемии для тяжести течения синдрома МОБ и обосновывают необходимость заместительной терапии препаратами фосфора. С другой стороны, нельзя игнорировать побочный эффект от приема фосфора — нефрокальциноз. Принимая его во внимание, мы стремимся назначать фосфор в уверенности, что потенциальная польза от препарата перевесит значимость побочного эффекта. Это поднимает вопрос — какое снижение фосфора считать клинически значимым для инициации терапии препаратом фосфора. У взрослых гипофосфатемией считается снижение фосфора менее 0,8 ммоль/л. У детей уровень фосфора крови колеблется в разные периоды жизни и, оценивая его значение, необходимо принимать во внимание пол и возраст. При этом, по разным исследованиям, границы референсных значений различаются [31–33]. Хотя причиной гипофосфатемии является нарушение на уровне почечных канальцев, как видно из таблицы 5, повышенная экскреция фосфора с мочой при синдроме МОБ не сразу привела к снижению фосфора крови у девочки: индекс максимальной реабсорбции фосфатов у девочки варьирует от 71 до 80% с 5,8 года, тогда как гипофосфатемия по одному из трех приведенных вариантов нормы выявляется с 7,9 года и по двум из трех вариантов нормы — с 8,7 года. К сожалению, нами не было проведено изначальное УЗИ почек до начала терапии, и мы не можем судить о наличии эхо-признаков уплотнения чашечно-лоханочной системы до начала приема препарата, но в любом случае с дополнительным приемом фосфора повышается нагрузка на почечный фильтр и риск прогрессии признаков нефрокальциноза растет. Эти соображения привели к отмене терапии в 7,9 года. За время терапии с 5,8 до 7,9 года произошел один перелом левой бедренной кости, появились признаки сколиоза, прогрессировали очаги ФД черепа; при этом присутствовали другие факторы с доказанным негативным действием на состояние костной ткани — тяжелое течение ФД, СТГ-гиперсекреция — что делает затруднительной оценку вклада терапии фосфором в течение заболевания. Уровень фосфора у девочки продолжает снижаться без значимой динамики показателей реабсорбции фосфора, что, скорее всего, вновь поднимет вопрос о необходимости терапии в будущем.

## ЗАКЛЮЧЕНИЕ

Синдром МОБ — тяжелое мультисистемное заболевание, характеризующееся пятнами цвета кофе-с-молоком, ФД костей, автономной эндокринной гиперфункцией и рядом неэндокринных проявлений.

Первые компоненты синдрома МОБ могут возникать на 1-м году жизни, но диагностика заболевания при этом может быть отсрочена до появления специфических клинических проявлений, среди которых — преждевременное половое развитие волнообразного течения. При случайном выявлении одного характерного компонента следует проявить настороженность и оценить возможность наличия синдрома МОБ.

При выявлении синдрома МОБ необходимо составить индивидуальный план ведения пациента с регулярным скринингом для своевременной диагностики новых компонентов.

Каждое проявление заболевания протекает со своей степенью тяжести, когда одни компоненты требуют только наблюдения, другие поддаются поддерживающей/ блокирующей терапии. При тяжелом течении компонент заболевания может прогрессировать, несмотря на проводимые меры по лечению и профилактике.

ФД при тяжелом прогрессирующем течении обуславливает рецидивирующие переломы и деформацию костей с риском развития значимых функциональных нарушений.

Преждевременное половое развитие при синдроме МОБ носит периферический характер на фоне эстроген-секретирующих кист. Для объективной оценки активности пППР следует проводить регулярное УЗИ малого таза. При частых рецидивах кист с прогрессией костного возраста и ухудшением ростового прогноза следует рассмотреть вопрос о назначении антиэстрогенной терапии.

Одним из вариантов патологии щитовидной железы при синдроме МОБ может быть многоузловой зоб с формированием множественных кистозных образований, который в отсутствие признаков злокачественности и значимого нарастания объема, как правило, позволяет придерживаться наблюдательной тактики годами.

СТГ-гиперсекреция требует назначения терапии аналогами соматостатина с коррекцией дозы при признаках декомпенсации для возможного предупреждения прогрессии очагов ФД черепа.

Гиперфосфатурия при синдроме МОБ может длительное время сопровождаться нормофосфатемией, вопрос о сроках назначения фосфора решается индивидуально; перед инициацией терапии следует проводить исследование почек для оценки потенциальной пользы и вреда планируемого лечения.

Многообразие клинических проявлений синдрома и особенности их течения в динамике определяют необходимость индивидуального подхода к терапии и наблюдению пациентов с синдромом МОБ.

## ДОПОЛНИТЕЛЬНАЯ ИНФОРМАЦИЯ

Источники финансирования. Аналитическая работа авторов выполнена по инициативе авторов без привлечения финансирования.

Конфликт интересов. Авторы декларируют отсутствие явных и потенциальных конфликтов интересов, связанных с содержанием настоящей статьи.

Участие авторов. Гирш Я.В. — существенный вклад в концепцию исследования, написание статьи; Карева М.А. — существенный вклад в концепцию и дизайн исследования, внесение в рукопись существенной правки с целью повышения научной ценности статьи; Маказан Н.В. — существенный вклад в концепцию и дизайн исследования, внесение в рукопись существенной правки с целью повышения научной ценности статьи; Давыгора Е.Н. — существенный вклад в концепцию исследования, написание статьи. Все авторы одобрили финальную версию статьи перед публикацией, выразили согласие нести ответственность за все аспекты работы, подразумевающую надлежащее изучение и решение вопросов, связанных с точностью или добросовестностью любой части работы.

Согласие пациента. Законный представитель пациента добровольно подписала информированное согласие на публикацию персональной медицинской информации в обезличенной форме.
